# Absolute Alcohol as a Disinfectant for Surgical Instruments

**Published:** 1897-04

**Authors:** 


### Absolute Alcohol as a Disinfectant for Surgical Instruments.

That absolute alcohol is not an absolute germicide is admitted, but that it has an area of usefulness in the care of instruments has been claimed. Dr. R. L. Randolph, in the *Johns-Hopkins Hospital Bulletin*, states that his experiments, as to the effect of this substance upon the keen-edged instruments of the ophthalmic surgeon, have led him to prefer it to the use of heat. He is satisfied that dry heat, as well as moist heat, dulls the edges of instruments. He says: "For the past eight years I have employed absolute alcohol as a disinfectant for all cutting instruments used in operations upon the eye. and recently I instituted a series of bacteriologic experiments to test the value of this agent as a practical disinfectant. The cataract operation demands a keener knife, probably, than any operation in surgery, and the peculiar objections to heat for sterilizing cutting instruments led me to adopt the use of absolute alcohol as the best substitute for heat." The alcohol employed in these experiments was Squibb's Absolute Alcohol, which is supposed to have a strength varying from $98\frac{1}{8}$ to 99.9 percent. This is the grade of alcohol which he uses in his operations. He presents the following conclusions: 1. That in a given number of eye instruments, by far the majority are infected by exposure to the air. 2. That absolute alcohol will seem a valuable disinfectant for instruments infected under the conditions which ordinarily surround us in every-day life. This conclusion seems warranted by the results obtained in the first and second series of experiments. Attention may be called to the fact, too, that in the second series the nails were all, without a doubt, infected, and it might be said that they had been exposed to conditions which, to say the least, were extraordinarily favorable for infection; so that this series, I think, is strongly suggestive that alcohol possesses disinfectant properties of no little value. 3. That the septic character of instruments infected with a pure culture of staphylococcus albus is not altered by exposure for twenty minutes to the action of absolute alcohol.

